# No Significant Effect of Coulomb Stress on the Gutenberg-Richter Law after the Landers Earthquake

**DOI:** 10.1038/s41598-020-59416-2

**Published:** 2020-02-19

**Authors:** Víctor Navas-Portella, Abigail Jiménez, Álvaro Corral

**Affiliations:** 1 Centre de Recerca Matemàtica, Edifici C, Campus Bellaterra, E-08193 Barcelona, Spain; 2 Barcelona Graduate School of Mathematics, Edifici C, Campus Bellaterra, E-08193 Barcelona, Spain; 3 Facultat de Matemàtiques i Informàtica, Universitat de Barcelona, Barcelona, Spain; 4 Departamento de Computación e Inteligencia Artificial, Universidad de Granada, Campus Ceuta, Cortadura del Valle s.n., E-51001 Ceuta, Spain; 5 Departament de Matemàtiques, Universitat Autònoma de Barcelona, E-08193 Barcelona, Spain; 6 Complexity Science Hub Vienna, Josefstädter Straße 39, 1080, Vienna, Austria

**Keywords:** Geophysics, Seismology

## Abstract

Coulomb-stress theory has been used for years in seismology to understand how earthquakes trigger each other. Whenever an earthquake occurs, the stress field changes, and places with positive increases are brought closer to failure. Earthquake models that relate earthquake rates and Coulomb stress after a main event, such as the rate-and-state model, assume that the magnitude distribution of earthquakes is not affected by the change in the Coulomb stress. By using different slip models, we calculate the change in Coulomb stress in the fault plane for every aftershock after the Landers event (California, USA, 1992, moment magnitude 7.3). Applying several statistical analyses to test whether the distribution of magnitudes is sensitive to the sign of the Coulomb-stress increase, we are not able to find any significant effect. Further, whereas the events with a positive increase of the stress are characterized by a much larger proportion of strike-slip events in comparison with the seismicity previous to the mainshock, the events happening despite a decrease in Coulomb stress show no relevant differences in focal-mechanism distribution with respect to previous seismicity.

## Introduction

Since the L’Aquila event in 2009 seismologists have advocated the modeling and testing of earthquakes within a rigorous statistical framework^1^, following on the CSEP (Collaboratory for the Study of Earthquake Predictability) previous works. A recent pseudo-prospective forecast was conducted on the 2010–2012 Canterbury, New Zealand, series, in order to test a total of fourteen earthquake models^2,3^. Its results offer some encouragement for a physical basis in earthquake forecasting and suggest that some of the recent physics-based and hybrid model development have added informative components^4^.

Our basic understanding of earthquake physics is that stress is being accumulated on certain regions due to different mechanisms, and that those regions rupture whenever that stress surpasses the strength of the material. That rupture is the earthquake. The mechanisms by which stresses change are diverse: in addition to tectonic driving, they can be induced by precedent earthquakes^5–9^, by volcanic activity^10^, or even by artificial means, such as injection of fluids^11^ or aquifer withdrawal^12^. Coulomb-stress theory has been used to forecast spatial patterns of aftershock rates, as well as assessing the likelihood of earthquake rupture sequences^13,14^. Although there exist instances where its predictive skills are arguable^15–18^, the monitoring of the changes in the stress field represents a valuable information for seismic and volcanic hazard forecasting and to proposing the adequate mitigation measures.

A hallmark of statistical seismology and of earthquake hazard assessment is the well-known Gutenberg-Richter relation, or Gutenberg-Richter law^19–21^. This law states that earthquake magnitudes must be described in terms of a probability distribution and that, above a lower cut-off value, this distribution is exponential. In terms of the probability density *f*(*m*) one has

$$f(m)=(b{\rm{ln}}10)1{0}^{-b(m-{m}_{min})}\propto 1{0}^{-bm},$$

defined for *m* ≥ *m*_*min*_ (values below *m*_*min*_ are disregarded), with *m* the magnitude, *m*_*min*_ the lower cut-off in magnitude, *b* the so called *b*-value (directly related to the exponent *β* of the power-law complementary cumulative distribution of seismic moment, *β* = 2*b*/3), and the symbol ∝ denoting proportionality. This relation is usually called magnitude-frequency distribution in seismology. A straightforward property of the exponential distribution leads to the fact that the rate (the number per unit time of earthquakes above a certain magnitude *m*) is also a decreasing exponential function of the magnitude, with the same *b*-value.

Earthquake hazard forecasts usually comprise two stages: in the first one, the rate of events is forecasted, while in the second one, the Gutenberg-Richter law is applied to those rates in order to obtain the probabilities of occurrence for each magnitude threshold. In the case of physics-based models, the forecasted rates of events depend on the Coulomb stresses calculated in the region of interest. These models are variants of the rate-and-state model by Dieterich^22^,

1 $$R(t)=r{\left[1+\left({e}^{-\Delta CFS/B}-1\right){e}^{-t/{t}_{a}}\right]}^{-1}$$

where *R*(*t*) is the rate of events (i.e., aftershocks) at any given time *t* after a mainshock, *r* is the rate of background seismicity, Δ*CFS* is the increase in Coulomb stress induced by the mainshock, *B* is a constant, for our purposes, and *t*_*a*_ is the characteristic relaxation time^22^.

Note that in the application of the Gutenberg-Richter law to the forecasted rate *R*(*t*) given by the previous expression it is implicit that the Coulomb-stress change caused by a mainshock does not alter the fulfillment of the Gutenberg-Richter law for the aftershocks, in particular, this law remains the same no matter whether Δ*CFS* is positive or negative. In some sense, *R*(*t*) inherits the dependence of the background rate *r* with the magnitude. Therefore, the rate-and-state formulation^14,22–26^ assumes the fulfillment of the Gutenberg-Richter law for the incoming events (aftershocks), with no change in the *b*-value. This assumption is made when inverting earthquake rates to obtain stress changes^10,27,28^. Physics-based models also assume the magnitude distribution does not depend on the stress values, so that forecasted rates can be translated into probabilities of occurrence for different magnitudes.

In fact, it has been long debated^29^ whether the value of *b* in the Gutenberg-Richter law is essentially universal^21^ or whether, on the contrary, it is affected by different geophysical conditions^30–32^. Some studies^30,31^ have correlated the *b*-value (and also the parameters of the Omori law^33–35^) with the style of faulting^36^. These studies indicate that (at least for California, for a long time period) *b* ≈ 1.03 for normal events, *b* ≈ 0.87 for strike-slip events, and *b* ≈ 0.79 for thrust events^30^. As the *b*-value is directly related to the log-ratio between the number of small and large earthquakes, variations in *b* can be associated with the ability of an earthquake rupture to propagate (more large events, low *b*) or not (less large events, high *b*).

According to Mohr-Coulomb theory^31,37^, thrust faults rupture at much higher Coulomb stress than normal faults (with strike-slip faults in between, assuming the same value for the coefficient of static friction). When the stress required to initiate a rupture is higher, stress interactions are enhanced and cracks can propagate faster in many different directions, yielding larger earthquakes^31^, consistent with the empirically observed *b*-values for thrust faulting^30^. Conversely, for lower rupture thresholds, one should find indeed the large *b*-values characterizing normal faulting. Although the threshold for triggering might be different for the different styles of faulting, the rupture or not of a fault also depends on its previous state.

Here we investigate, with rigorous statistical tools, if the Gutenberg-Richter law is affected by the binary choice between positive and negative increases of the Coulomb stress, using the sequence of aftershocks after the 1992 Landers earthquake. This event is chosen for illustration due to its particular relevance, as it yields one of the most studied mainshocks and aftershock sequences in the history of seismology; nevertheless, we are equally interested in developing the methodological aspects to approach this problem. The next section explains the seismic catalog and the spatio-temporal window used to define this sequence. Section 3 develops the procedure to calculate the increase in the Coulomb stress that the Landers earthquake provokes in the fault plane of each event in the sequence. The statistical analysis is also exposed in this section. Section 4 presents the results and section 5 summarizes the conclusions. We anticipate that the number of Landers aftershocks in the relevant window of space, time, magnitude, and Coulomb-stress increase is too low to reach statistically significant results. We suspect this may be a general characteristic of many other important aftershock sequences.

## Data

The June 28, 1992, Landers earthquake, with a moment magnitude *m* = *M*_*w*_ = 7.3 and a rake angle *ρ* = −177°, corresponding to strike-slip focal mechanism, has been the strongest one in Southern California at least since 1952. The earthquake and its subsequent aftershock sequence have been extensively studied^38–40^, with a number of slip distributions that describe its rupture^41–44^. In this work we use four slip models to calculate the strain; these models are: Wald and Heaton (referred here to as **wald**)^41^, Hernandez *et al*. (**hernandez**)^42^, Landers Big-Bear California (**bbcal**)^43^ and Landers Surface Rupture (**surfrup**)^44^. The terminology is the same as the one used in ref. ^43^.

High quality catalogs for Southern California are nowadays available^45,46^; in particular in this paper we will select the Landers’ aftershocks from the Yang-Hauksson-Shearer (YHS) catalog^47^, which incorporates focal-mechanism solutions. Given the distribution of acceptable mechanisms, the preferred solution is the most probable one^48^. The ambiguity of the actual fault plane is solved by considering that the preferred nodal planes are those associated with the preferred solution listed in the catalog^47^. The focal mechanism, in concrete, the rake angle, together with Landers stress field derived from the slip model, allows us to calculate Coulomb-stress increases (positive or negative) induced by the mainshock on the actual orientations of the aftershock fault planes. Nevertheless, if one does not trust the preferred solutions reported in the catalog, an alternative procedure can be applied based on selecting the nodal plane in which the Coulomb stress increase is maximum^49^. Note that the YHS catalog does not report the moment magnitude necessarily but a preferred magnitude.

In order to better detect the influence of the Landers stress change we take a time window of 100 days after the mainshock and a spatial window going from 10 to 150 km from the mainshock rupture. The time of occurrence of the Landers earthquake is taken as the time origin except for the bbcal slip model where the time origin is set by the Big-Bear earthquake (which occurred approximately three hours after Landers earthquake with a moment magnitude *m* = *M*_*w*_ = 6.3 and rake angle *ρ* = −180°^43^). We tried other choices for the limits of the window finding similar results as reported in the Supplementary Material. The referred spatio-temporal window defines Landers aftershocks for our purposes. Distances to the Landers rupture are computed as the minimum Euclidean distance from the aftershock hypocenter to the center of each rupture patch as given by the slip model. The reason to exclude events closer than 10 km is the uncertainty of the deformation field near the edges of the subfaults^50^, as the finite-fault approximation provides spurious values near the fault zone because of boundary effects.

## Procedure

### Continuum mechanics

The dMODELS software in ref. ^51^ calculates the deformation field (or displacement) caused by different models corresponding to different physical processes. Although there exist many programs that calculate deformation caused by earthquakes, this package has been thoroughly tested, and can introduce many different sources of deformation, which can be translated into stress changes in a straightforward way. The dMODELS software will be the one used here to obtain deformation field from the different slip models of Landers.

The local coordinate system for dMODELS is east-north-up, ENU. After introducing the corresponding slip model (also called source model) for the mainshock of interest (Landers in our case^43^) into the dMODELS program we obtain the projections in the ENU axes of the deformation field $\overrightarrow{u}$ caused by the mainshock at the position of each aftershock (and also at its neighborhood, in order to take spatial derivatives). We then obtain the strain tensor associated to $\overrightarrow{u}$ by calculating the (symmetrized) gradient of the deformation^52^, whose components are *ε*_*ij*_ = (∇_*i*_*u*_*j*_ + ∇_*j*_*u*_*i*_)/2 (with a spatial step equal to 1 km).

Afterwards, we assume an isotropic and elastic material for calculating the stress tensor^52^, or, more precisely, the contribution of the mainshock to the stress tensor, *s*_*ij*_ = 2*με*_*ij*_ + *λδ*_*ij*_∑_*k*_*ε*_*kk*_, with *δ*_*ij*_ the components of the identity matrix and with the Lamé elastic moduli given by *μ* = *λ* = 3 × 10^4^ MPa^37^ (Poisson ratio $\nu =\lambda {\left(\lambda +\mu \right)}^{-1}/2=0.25$). Moreover, when calculating the stress induced by previous events (mainshocks) on new events (aftershocks) it is necessary to orientate it onto the aftershock fault planes^53,54^, so that one can actually evaluate if the new events could have been triggered by the induced stress or not. Given the fault plane and slip vector of an aftershock, we calculate the change in the normal *σ*_*n*_ and shear (or tangential) *τ* stresses in that orientation and position, as

2 $$\Delta {\sigma }_{n}={\sum }_{ij}{n}_{i}{s}_{ij}{n}_{j}\,{\rm{and}}\,\Delta \tau ={\sum }_{ij}{\ell }_{i}{s}_{ij}{n}_{j},$$

with *n*_*i*_ and *ℓ*_*i*_ the components of the normal and slip vectors, respectively. The formulas to obtain the ENU components of these vectors from the information recorded in the YHS catalog (strike, dip and rake angles^55^) are given in the Methods section. Our calculation of the Coulomb-stress changes over the planes of the actual faults^53^ is in contrast with an approach in which Coulomb stresses are calculated onto the so-called optimally oriented planes^6^, when the only information available is the regional stress. However, optimally oriented planes are imaginary planes that might not correspond to the actual geology, and thus, our approach is more realistic.

The Mohr-Coulomb failure criterion^56^ states that the shear stress *τ* on a fault that ruptures must surpass the critical value *τ*_*c*_, which is a linear function of the normal stress,

3 $${\tau }_{c}=C-\mu {\prime} {\sigma }_{n}$$

with *C* the cohesion and $\mu {\prime}$ the effective fault friction coefficient (including the contribution of the pore pressure^6,57^). Care must be taken with the convention of signs in the normal stress, which is not the same in geophysics than in solid mechanics (our convection takes the negative sign for compression, this is the reason for the negative sign before $\mu {\prime}$). From this failure criterion it is natural to define the Coulomb stress as

4 $$CFS=\tau +\mu {\prime} {\sigma }_{n},$$

which signals failure by *CFS* > *C*. In fact, for pre-existing faults one can consider that the cohesion is nearly zero. In any case, the change in Coulomb stress at the aftershock fault plane due to the mainshock will be

5 $$\Delta CFS=\Delta \tau +\mu {\prime} \Delta {\sigma }_{n},$$

with Δ*τ* and Δ*σ*_*n*_ coming from Eq. (2). Thus, positive increases of the Coulomb stress bring the fault closer to failure, whereas negative increases distance it away from failure. As the real value of the effective friction coefficient ${\mu }^{{\prime} }$ is uncertain^37^, we will check different values of it as in ref. ^15^.

**Figure 1:**
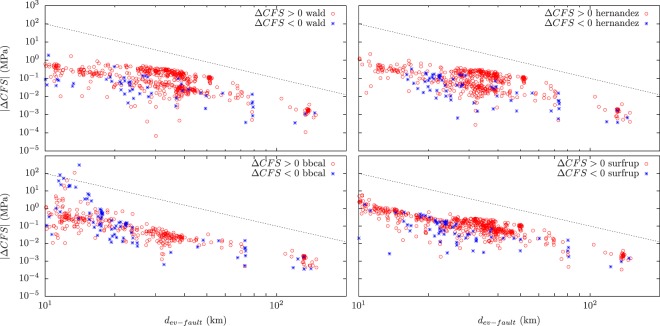
Dependence of the absolute value of the change in the Coulomb stress *ΔCFS* as a function of the distance of the aftershocks to the Landers rupture for each slip model, with $\mu {\prime} =0.4$ and *m* ≥ 3. Aftershocks correspond to the first 100 days after the mainshock and distance is restricted to the range from 10 to 150 km. Black dashed line with slope −3, as stated by Coulomb theory, is shown as a guide to the eye.

**Figure 2:**
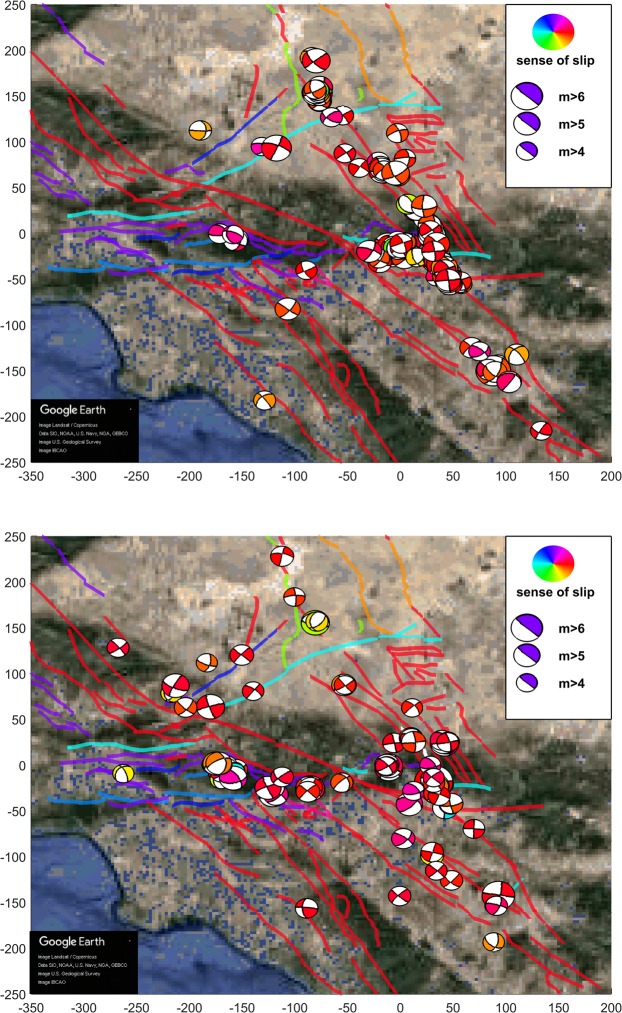
Focal mechanism representation for strike-slip Landers aftershocks, separated in terms of Δ*CFS*, as calculated from the hernandez slip model (time window of 100 days after the mainshock). Top: Δ*CFS* > 0. Bottom: Δ*CFS* < 0. Color scale represents the sense of slip (rake) and fault traces are also shown using the same color code: red for right-lateral (*ρ* close to ±180°), light blue for left-lateral (*ρ* close to 0°), green for normal (*ρ* close to −90°) and dark blue or purple for thrust faulting (*ρ* close to 90°). An area of 550 × 500 km is shown; aftershocks are restricted to *m* ≥ 4 (for clarity sake). Aftershocks beyond the limit of 150 km are also shown. Both axes display distances with respect an arbitrary origin, in km.

**Figure 3:**
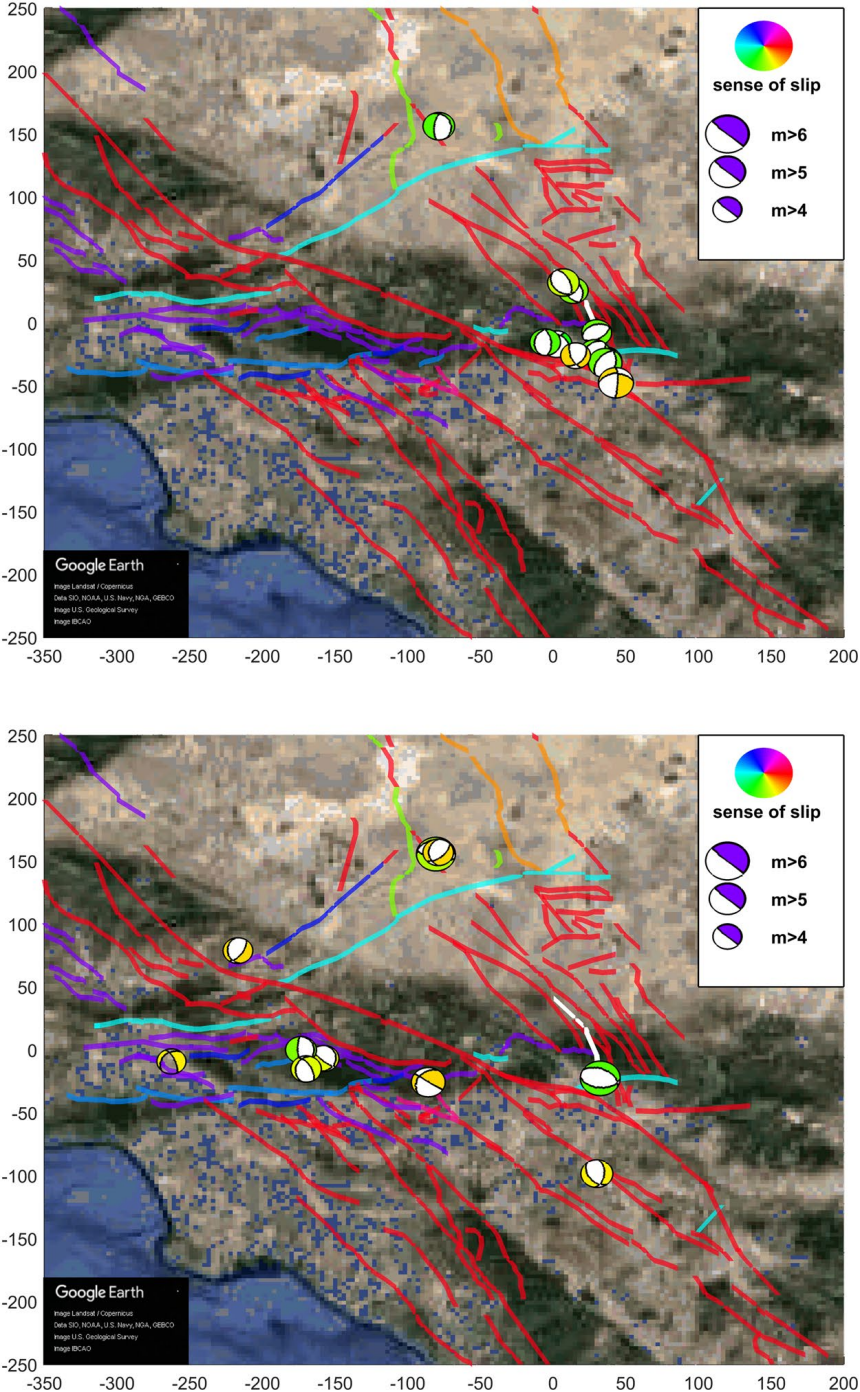
Same as previous figure, for aftershocks with normal focal mechanism.

**Figure 4:**
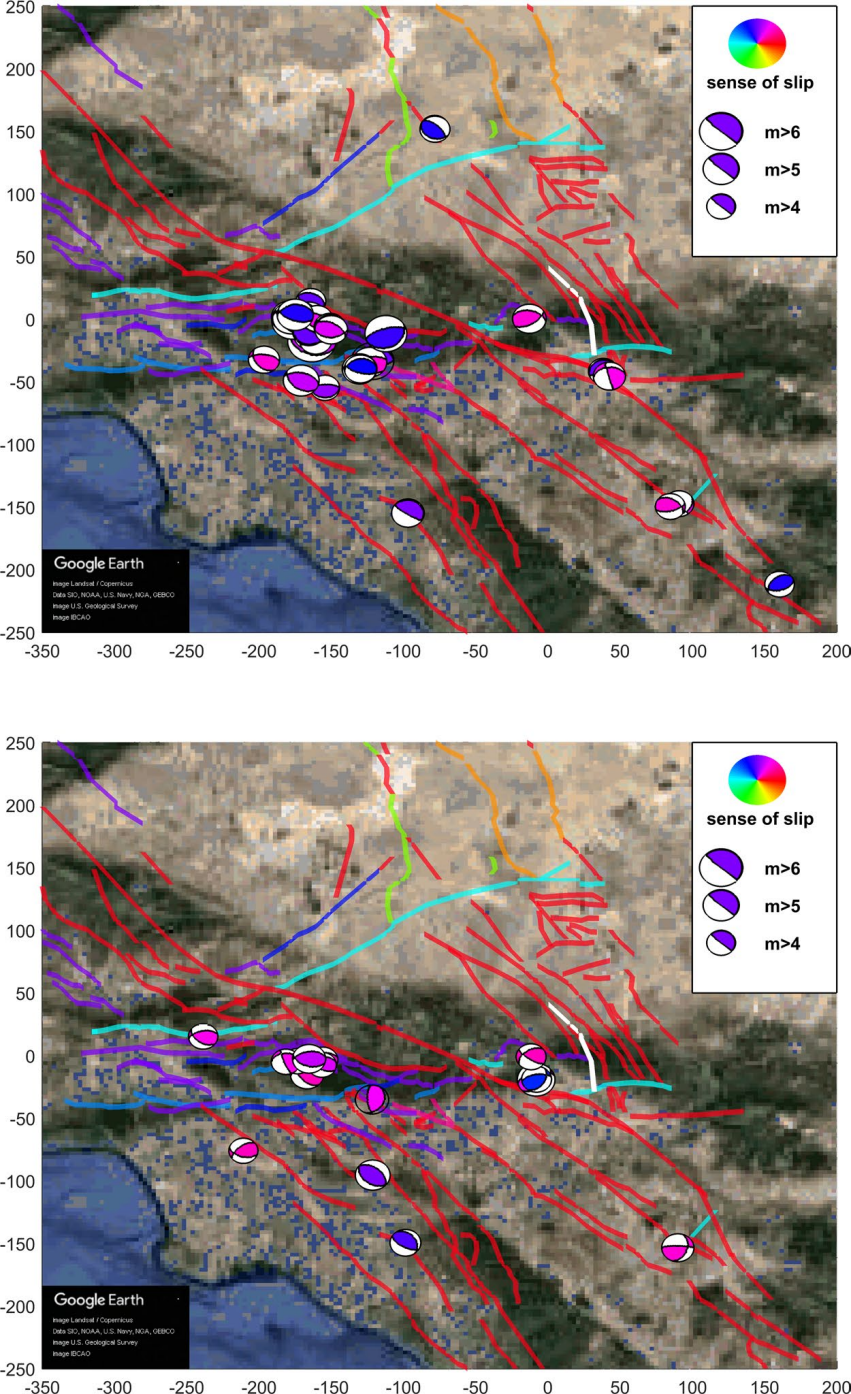
Same as previous figure, for aftershocks with thrust focal mechanism.

In Fig. 1 we present a scatter plot that shows the resulting absolute values of the increase of Coulomb stress |Δ*CFS*| over each aftershock and their dependence with the distance between the aftershock and the Landers rupture for the four slip models. As it is implicit by the Coulomb theory, the value of the increase of Coulomb stress decays as the cube of the distance to the rupture. In Figs. 2, 3 and 4 we show aftershocks with strike-slip, normal and thrust focal mechanisms respectively for *m* ≥ 4 and positive and negative Δ*CFS*, as calculated from the hernandez slip model. The same figures including aftershocks with magnitude larger than 3 are shown in the Supplementary Material.

### Statistical methods

Once we know the Coulomb-stress change in the fault plane of each aftershock we can separate these into two subsets attending to the value of the change, with the most natural separation being between positive and negative increases (denoted by sub-indexes > and < , respectively). Naturally, we expect to obtain many more aftershocks with positive Δ*CFS* than with negative Δ*CFS*^58^. It is for each of these subsets that we will study the fulfillment of the Gutenberg-Richter law. For any set or subset (or sub-catalog) of earthquakes, the value of *b* in the Gutenberg-Richter law can be automatically obtained by maximum-likelihood estimation, as^59,60^:

6 $$b=\frac{{{\rm{\log }}}_{10}e}{\bar{m}-{m}_{min}},$$

with $\bar{m}$ the mean magnitude of the events considered (i.e., those above *m*_*min*_). Let us stress that *m*_*min*_ is not the minimum magnitude recorded in the catalog but the value from which we fit the Gutenberg-Richter law to the data. As the resolution of the magnitude Δ*m* is small (Δ*m* = 0.01) it is not necessary to perform the discreteness correction^61^.

In principle, results should not significantly depend on the value of *m*_*min*_, but the larger its value the less data to calculate the *b*-value and the larger the uncertainty, whereas for a too small *m*_*min*_ the Gutenberg-Richter law would not be fulfilled due to the incompleteness of the catalog and the resulting *b*-value would be artefactual. In this paper we have taken *m*_*min*_ = 3, which ensures the fulfillment of the Gutenberg-Richter law for all data sets analyzed, as we have verified by means of the Kolmogorov-Smirnov goodness-of-fit test^62^, where the distribution of the test statistic and, from it, the *p*-value of the fit, *p*_*fit*_, is calculated using 10^4^ Monte Carlo simulations^63,64^. Although some fitting procedures look for the value of *m*_*min*_ that optimizes the fit for a given data set^63–65^, we have opted for a fixed *m*_*min*_ in order to compare the different subsets on the same footing. So, in all cases the exponential fit for *m* ≥ 3 cannot be rejected (*p*_*fit*_ larger than 0.05). Note that *m*_*min*_ defined in this way can be considered a magnitude of completeness, and thus, our value of *m*_*min*_ turns out to be rather conservative or strict, in the sense that it is larger (and therefore safer) than in other works^66^.

The maximum-likelihood estimation of the *b*-value has an associated uncertainty given by its standard deviation

$$\sigma =\frac{b}{\sqrt{N}},$$

where *N* is the number of earthquakes with *m* ≥ *m*_*min*_ in the subset, out of a total number *N*_*tot*_ (of any magnitude)^67^. Note that this uncertainty only depends on the number of data, and has nothing to do with the goodness of the fit. This result, as well as the formula for the maximum-likelihood estimation of *b*, Eq. (6), can also be obtained from ref. ^64^ just taking into account the relation between moment magnitude and seismic moment. The standard deviation, *σ*, is what represents the uncertainty when we report our resulting *b*-values.

The comparison between the *b*-values of the subsets with different values of *ΔCFS* is done by means of the following statistic

$$z=\frac{{b}_{ > }-{b}_{ < }}{\sqrt{{\sigma }_{ > }^{2}+{\sigma }_{ < }^{2}}}=\frac{{b}_{ > }-{b}_{ < }}{\sqrt{{b}_{ > }^{2}/{N}_{ > }+{b}_{ < }^{2}/{N}_{ < }}},$$

where the sub-indexes > and < refer to positive and negative increases of the Coulomb stress. This statistics is rooted on the null hypothesis that both subsets of data (positive and negative) belong to the same underlying population of earthquake magnitudes and then, both estimators of the *b*-value (*b*_>_ and *b*_<_) have a common mean value, which is that of the whole population. Therefore, under the null hypothesis, *b*_>_−*b*_<_ has zero mean and standard deviation $\sqrt{{\sigma }_{ > }^{2}+{\sigma }_{ < }^{2}}$ (approximating the population variance from the sample values of *b*_>_ and *b*_<_ and assuming zero covariance between *b*_>_ and *b*_<_) and then *z* has zero mean too and unit standard deviation. An additional assumption is that *z* is normally distributed, which is supported by theory in the asymptotic limit (*N*_>_ and *N*_<_ going to infinity^68^). Assuming normality we will test the null hypothesis just comparing the value of *z* with the standard normal distribution and the hypothesis will be rejected if the value of *z* is too extreme for a given significance level; in quantitative terms this will be given by a *p* − value, called *p*_*norm*_, smaller than the significance level (0.05, let us say; corresponding to 0.95 confidence).

If we do not want to believe that the asymptotic regime has been reached the best option is to use a permutation test^69^. Under the null hypothesis (same underlying population of magnitudes) one is allowed to aggregate both subsets (positive and negative) and take, without repetition, two sub-samples of size *N*_>_ and *N*_<_; note that this is equivalent to take a permutation of the aggregated sample and separate it into two parts (> and <). One proceeds in the same way as in the original data, calculating (by maximum likelihood) ${b}_{ > }^{\ast }$, ${b}_{ < }^{\ast }$, and from here ${\sigma }_{ > }^{\ast }$, ${\sigma }_{ < }^{\ast }$, and z^*^, where the asterisk marks that we are dealing with a permutation of the original data. Repeating the permutation procedure many times we find the distribution of z^*^, which can be compared with the original value *z*. The *p* − value of the permutation test, *p*_*perm*_, will be given by the fraction of permutations for which |z^*^| is larger than |*z*| (the empirical value). In our case we take 10^4^ permutations.

As a complement, instead of the fitted *b*-values we may directly compare the distributions; this can be done with the two-sample Kolmogorov-Smirnov test, whose null hypothesis is that both data sets come from the same population, so, the two empirical distributions (> and <) are two realizations of a unique theoretical distribution (which remains unveiled)^62^. This test leads to a *p*-value that we call *p*_2*ks*_. A final comparison comes from the application of the Akaike information criterion (*AIC*)^70^. We consider that we aggregate both subsets (positive and negative *ΔCFS*) but keeping the distinction in the sign of Δ*CFS*. Then, we contemplate two options. Model 1, simple: we fit the aggregated data set with one single Gutenberg-Richter exponential leading to the value *b*_*all*_. Model 2, “complex”: we fit each data set with its own exponential function (values *b*_>_ and *b*_<_). In each case, $AIC=2k-2\widehat{\ell }$, where *k* is the number of parameters of each model and $\widehat{\ell }$ is the log-likelihood of the model at maximum. The likelihood in model 2 is the sum of likelihoods for each subcatalog^68^. The model yielding the smallest *AIC* should be prefered. Defining Δ*AIC* = *AIC*_2_ − *AIC*_1_ leads to the rejection of the simple model when Δ*AIC* is significantly below zero (see next section).

## Results

 Table 1 shows the values of *b* obtained from the application of the maximum-likelihood-estimation procedure explained above to the different subcatalogs obtained from the Landers sequence. We can see how, in the overall case (when events are not separated in terms of Coulomb-stress change), the Gutenberg-Richter law is fulfilled with an average value *b*_*all*_ = 0.92. Each slip model leads to a different value of *b*_*all*_ because the fault geometry is different, and events too close to the fault are discarded. This *b*-value for the Landers aftershocks is found, not surprisingly, to be close to the average for aftershocks in California, *b* ≃ 0.9^71,72^, and somewhat below the long-term value of Southern California (all events), *b* ≃ 1.0^73^ (although other works report *b* ≃ 1.0 for Landers aftershocks, probably due to the consideration there of a much smaller magnitude of completeness^74^).

**Table 1:** Results of fitting the Gutenberg-Richter law to the Landers aftershocks, separating positive and negative Coulomb-stress increases, for different slip models, $\mu {\prime} =0.4$, and *m*_*min*_ = 3. Aftershocks correspond to the first 100 days after the Landers mainshock and their distance to the Landers rupture is restricted to be between 10 and 150 km. The *p*-value of the goodness-of-fit test is computed with 10^4^ simulations and is denoted by *p*_*fit*_. Its uncertainty corresponds to one standard deviation. In no case the Gutenberg-Ricther law can be rejected.

Slip model		*N*_*tot*_	*N*	*b*-value	*σ*	*p*_*fit*_
**wald**	Δ*CFS* > 0	5213	509	*b*_>_ = 0.927	0.041	0.313 ± 0.005
	Δ*CFS* < 0	814	51	*b*_<_ = 0.766	0.107	0.861 ± 0.003
	All	6027	560	*b*_*all*_ = 0.909	0.038	0.243 ± 0.004
**hernandez**	Δ*CFS* > 0	5027	465	*b*_>_ = 0.926	0.043	0.505 ± 0.005
	Δ*CFS* < 0	765	62	*b*_<_ = 0.866	0.110	0.197 ± 0.004
	All	5792	527	*b*_*all*_ = 0.919	0.040	0.231 ± 0.004
**bbcal**	Δ*CFS* > 0	3641	309	*b*_>_ = 0.978	0.056	0.232 ± 0.004
	Δ*CFS* < 0	1191	82	*b*_<_ = 0.948	0.105	0.327 ± 0.005
	All	4832	391	*b*_*all*_ = 0.971	0.049	0.053 ± 0.002
**surfrup**	Δ*CFS* > 0	5534	548	*b*_>_ = 0.890	0.038	0.290 ± 0.005
	Δ*CFS* < 0	774	68	*b*_<_ = 0.891	0.108	0.555 ± 0.005
	All	6308	616	*b*_*all*_ = 0.890	0.036	0.239 ± 0.004

### Comparison of distributions

After separating by the sign of the Coulomb-stress change, the first result that becomes apparent from Table 1 is that the number of aftershocks with positive increases is much larger than the number for the negative case^6,7^, no matter the slip model used to calculate Δ*CFS*. Regarding the *b*-values, although they depend on the slip model, we can summarize them by taking the mean of the four models, which, for $\mu {\prime} =0.4$, is *b*_>_ ≃ 0.93 and *b*_<_ ≃ 0.87 with individual uncertainties around 0.04 and 0.11 respectively. Note that the magnitude distribution for the overall case is a mixture of the distributions corresponding to Δ*CFS* > 0 and Δ*CFS* < 0, and therefore, the value of *b* in the overall case turns out to be the harmonic mean of *b*_>_ and *b*_<_, i.e.,

7 $${b}_{all}^{-1}=\frac{{N}_{ > }{b}_{ > }^{-1}+{N}_{ < }{b}_{ < }^{-1}}{{N}_{ > }+{N}_{ < }},$$

see refs. ^75,76^. In order to properly compare the values of *b*_>_ and *b*_<_, statistical testing becomes necessary^77^.

**Figure 5:**
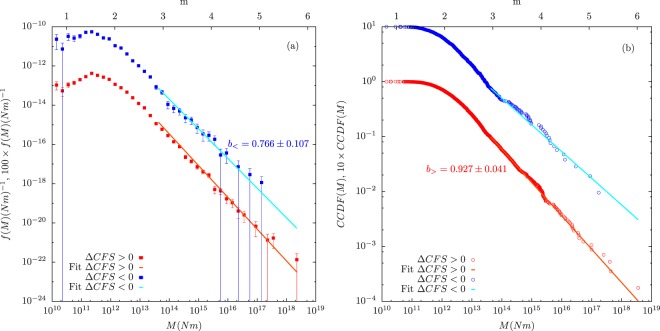
Estimation of the probability densities (**a**) and of the complementary cumulative distribution functions (CCDF) (**b**) of seismic moment *M* separating in terms of *ΔCFS* > 0 and *ΔCFS* < 0 for Landers aftershocks during 100 days using the wald slip model and $\mu {\prime} =0.4$. Curves corresponding to *ΔCFS* < 0 have been conveniently multiplied by a factor 100 and 10, respectively, for clarity sake. Error bars in (**a**) denote one standard deviation, and are symmetric, despite the appearance in log scale, see ref. ^64^. Correspondence between seismic moment *M* and magnitude *m* is also provided.

 Table 2 compares *b*_>_ and *b*_<_ for the different slip models taking $\mu {\prime} =0.4$, and shows that the difference in the *b*-values can not be considered significantly different from zero with a confidence larger than 0.95 so, the null hypothesis *b*_>_ ≃ *b*_<_ can not be rejected. This result is true for all the statistical tests as all the *p*-values (*p*_*norm*_ and *p*_*perm*_) are greater than 0.05. Table 2 also shows the results of the two-sample Kolmogorov-Smirnov test and the calculation of Δ*AIC* leading in both cases to the result that no change in the distributions as a function of positive and negative Δ*CFS* can be established. In concrete, Δ*AIC* is always greater than the critical value Δ*AIC*_*c*_ = −1.84^70,78^ at significance level of 0.05, so the simple model with a unique exponent *b*_*all*_ is preferred. The wald slip model is the one for which both distributions (positive and negative) appear as more different; however, the difference is not significant. Figure 5 shows the probability density functions as well as the complementary cumulative distribution functions in this case. Similar results are found when, instead of using the solutions provided by the YHS catalog, the nodal plane in which the Coulomb stress increase is maximum is selected for each aftershock^49^. The results obtained through this procedure are shown in the Supplementary Material.

**Table 2:** Results of the statistical tests comparing *b*-values and magnitude distributions for positive and negative Coulomb-stress changes, using different slip models and $\mu {\prime} =0.4$ (same data as previous table). Columns 2 to 4: testing the null hypothesis that there is no difference between the *b*-values (i.e., *b*_>_ = *b*_<_). Both asymptotic normality of the *z* statistic and a permutation test are used for the calculation of the *p*-value (labeled as *p*_*norm*_ and *p*_*perm*_, respectively). In the latter case the number of permutations is 10^4^, and the uncertainty of *p*_*perm*_ corresponds to one standard deviation. Columns 5 to 6: testing the null hypothesis that there is no difference in the distributions, using the 2-sample Kolmogorov-Smirnov test. *d*_2*ks*_ and *p*_2*ks*_ are the 2-sample Kolmogorov-Smirnov statistic and its *p*-value. Values of Δ*AIC* = *AIC*_2_ − *AIC*_1_ are also included in the last column.

Slip model	*z*	*p*_*norm*_	*p*_*perm*_	*d*_2*ks*_	*p*_2*ks*_	*ΔAIC*
**wald**	1.396	0.163	0.156 ± 0.004	0.139	0.311	0.234
**hernandez**	0.511	0.609	0.596 ± 0.005	0.095	0.690	1.748
**bbcal**	0.254	0.800	0.828 ± 0.004	0.063	0.952	1.936
**surfrup**	− 0.010	0.992	0.994 ± 0.001	0.094	0.643	1.999

### Influence of focal mechanism

As mentioned in the introduction, some authors have unveiled a direct dependence of the *b*-value on the focal mechanism of the events, which implies a dependence of *b* on the total stress (not the stress increase)^30^. The rake angle is associated to the focal mechanism in the following way: values of the rake around − 90° correspond to normal events (labelled as *no*), values around 0° or ± 180° to strike-slip events (*ss*), and values around 90° to thrust events (*th*). We do not find any significant effect of the rake on the *b*-value (see Table 3), due to the low number of events in the normal and thrust regimes (which increases the uncertainty). But despite the large uncertainty, the values of *b*_*no*_ and *b*_*ss*_ are roughly in agreement with the results of ref. ^30^; however, our value of *b*_*th*_ turns out to be rather large in comparison (similar to *b*_*ss*_ but compatible also with *b*_*no*_, within the error bars).

**Table 3:** Number of events and *b*-values corresponding to Landers aftershocks with *m* ≥ 3 separated by sign of the Coulomb-stress increase (> and <) and by focal mechanism (*fm*) for each slip model. *fm* = *no* (normal), *ss* (strike-slip), and *th* (thrust). The Coulomb stress is calculated with $\mu {\prime} =0.4$. Same data as in previous tables. Values of *b* calculated with 10 or less events are not reported. Values for the 5 years previous to Landers are also included and labelled by the superscript *pre*.

fm	*N*_>*fm*_	*N*_<*fm*_	*N*_*fm*_	${N}_{fm}^{pre}$	*b*_>*fm*_	*b*_<*fm*_	*b*_*fm*_	${b}_{fm}^{pre}$
**wald**								
No: − 135° ≤ *ρ* ≤ − 45°	39	5	*N*_*no*_ = 44	${N}_{no}^{pre}=77$	1.047	—	*b*_*no*_ = 1.081	${b}_{no}^{pre}=1.484$
Th: 45° ≤ *ρ* ≤ 135°	9	8	*N*_*th*_ = 17	${N}_{th}^{pre}=77$	—	—	*b*_*th*_ = 0.929	${b}_{th}^{pre}=0.899$
SS: the rest	461	38	*N*_*ss*_ = 499	${N}_{ss}^{pre}=503$	0.914	0.726	*b*_*ss*_ = 0.896	${b}_{ss}^{pre}=0.960$
**hernandez**								
No: − 135° ≤ *ρ* ≤ − 45°	38	3	*N*_*no*_ = 41	${N}_{no}^{pre}=78$	0.995	—	*b*_*no*_ = 1.011	${b}_{no}^{pre}=1.502$
Th: 45° ≤ *ρ* ≤ 135°	7	10	*N*_*th*_ = 17	${N}_{th}^{pre}=61$	—	—	*b*_*th*_ = 0.929	${b}_{th}^{pre}=0.899$
SS: the rest	420	49	*N*_*ss*_ = 469	${N}_{ss}^{pre}=506$	0.920	0.840	*b*_*ss*_ = 0.911	${b}_{ss}^{pre}=0.957$
**bbcal**								
No: − 135° ≤ *ρ* ≤ − 45°	22	4	*N*_*no*_ = 26	${N}_{no}^{pre}=80$	1.128	—	*b*_*no*_ = 1.165	${b}_{no}^{pre}=1.521$
Th: 45° ≤ *ρ* ≤ 135°	7	5	*N*_*th*_ = 12	${N}_{th}^{pre}=69$	—	—	*b*_*th*_ = 1.309	${b}_{th}^{pre}=0.831$
SS: the rest	280	73	*N*_*ss*_ = 353	${N}_{ss}^{pre}=507$	0.970	0.886	*b*_*ss*_ = 0.951	${b}_{ss}^{pre}=0.958$
**surfrup**								
No: − 135° ≤ *ρ* ≤ − 45°	46	5	*N*_*no*_ = 51	${N}_{no}^{pre}=76$	0.939	—	*b*_*no*_ = 0.966	${b}_{no}^{pre}=1.470$
Th: 45° ≤ *ρ* ≤ 135°	9	11	*N*_*th*_ = 20	${N}_{th}^{pre}=61$	—	1.010	*b*_*th*_ = 0.886	${b}_{th}^{pre}=0.899$
SS: the rest	493	52	*N*_*ss*_ = 545	${N}_{ss}^{pre}=504$	0.888	0.844	*b*_*ss*_ = 0.884	${b}_{ss}^{pre}=0.961$

We further observe that ratios *N*_>*ss*_/*N*_<*ss*_ and *N*_>*no*_/*N*_<*no*_ are higher than *N*_>*th*_/*N*_<*th*_; i.e., in strike-slip and normal events the contribution from Δ*CFS* > 0 is higher than in thrust events, as can be verified looking at Table 3. Comparing with the number of earthquakes with each focal mechanism for the 5 years previous to Landers we conclude that it is indeed the low number of thrust aftershocks with positive Δ*CFS* which is anomalous (and not the relatively high number of them for negative Δ*CFS*), due to an increase in the number of normal events and an even higher increase in strike-slip events triggered (Δ*CFS* > 0) by the Landers mainshock. This difference in numbers becomes visually apparent in Figs. 2, 3 and 4.

## Discussion

We have seen how the positive Coulomb-stress increase associated to the Landers mainshock triggered a very large number of strike-slip events and also a large number of normal events, but much less thrust events. Although this result seems easy to establish, as it can be obtained without the calculation of Δ*CFS* (due to the fact that most of the events have Δ*CFS* > 0 and thus, this subset dominates the overall statistics), we have unambiguously associated these events to the positive Δ*CFS*. On the other side, the events in the opposite regime (with Δ*CFS* < 0) keep a proportion between normal, strike-slip, and thrust events rather different to the Δ*CFS* > 0 case, and close to that of the immediately previous record (1987–1992, up to Landers). These results are largely independent on the slip model used to calculate the change in Coulomb stress. We have also found that the *b*-values of the Gutenberg-Richter law for which the Landers event yielded a positive Δ*CFS* (with *b*_>_ ≃ 0.93) are in general larger than the *b*-values for the events with negative Δ*CFS* (*b*_<_ ≃ 0.87); nevertheless, this difference is not statistically significant for any of the slip models used to compute the change in the Coulomb stress.

A non-significant result teaches us that the differences in *b*-values may be spurious and that certainly, more careful research is necessary in order to overcome statistical limitations. We urge the study of other aftershock sequences for which both knowledge of focal mechanisms as well as detailed slip models for the mainshock are available. It may happen, as for the Landers sequence, that the restrictions in space, time, magnitude, and Coulomb-stress change are too many to yield significant results (in our case, the restriction *m* ≥ 3 is particularly strong, but necessary for the fulfillment of the Gutenberg-Richter law). In such a case of low statistics, aggregation (stacking) of sequences from different mainshocks could reduce statistical uncertainty and lead to significant results. This is left for future research.

In addition, a number of extensions and improvements could be incorporated to our approach. We make use of slip models with relatively low resolution in space; so, it would be interesting to know if higher resolution slip models^79,80^ lead to somewhat different values of the strain and the stress, in particular close to the fault. Also, some authors have argued that real faults should have rather low values of the $\mu {\prime}$ coefficient^81^. We provide some check of this in the Supplementary Material, which leads to the conclusion that $\mu {\prime}$ has little influence on the *b*-values. Further, in our temporal window of 100 days, the effect of viscoelastic relaxation^82^ should be important; so, this would need to be incorporated into the calculation of the stress. Perhaps more relevant but easier to implement would be the contribution to the stress of the triggered events^83^. Knowing the focal mechanism of each of these events allows us to calculate the slip direction, which, together with an estimation of the slip value from the magnitude, can be considered an elementary slip model. From this, the event’s contribution to the strain can be computed in the same way as for the mainshock. Moreover, we could take into account the relation between *b*-values and differential stress^84^.

Finally, in a preliminary analysis we have seen that there is no substantial difference in the fulfilling of the Omori law^33–35^ in the two populations of events (Δ*CFS* > 0 and < 0). Indeed, if we compare this for the two subsets we find the "characteristic” power-law Omori decay of the rate with very similar values of the Omori exponent. Note that this is in disagreement with the rate-and-state formulation^22^, which does not predict Omori behavior in the case of negative Δ*CFS*, but instead, contemplates a sudden reduction of seismicity followed by a gradual recovery. The issues discussed in this paper about the Gutenberg-Richter law are equally important in relation to the Omori law. Further research in the lines we point to would be of great interest^85^.

## Methods

The YHS catalog characterizes fault planes and slip vectors by means of three angles: strike *Θ*, dip *δ*, and rake *ρ*. In term of these, the normal vector of the fault is given by

8 $$\widehat{n}=\left(\begin{array}{l}{n}_{E}\\ {n}_{N}\\ {n}_{U}\end{array}\right)=\left(\begin{array}{c}\cos \Theta \sin \delta \\ -\sin \Theta \sin \delta \\ \cos \delta \end{array}\right)$$

in the ENU coordinate system^86^. In the same way, the slip vector is obtained as

9 $$\widehat{\ell }=\left(\begin{array}{l}{\ell }_{E}\\ {\ell }_{N}\\ {\ell }_{U}\end{array}\right)=\left(\begin{array}{l}\sin \Theta \cos \rho -\cos \Theta \cos \delta \sin \rho \\ \cos \Theta \cos \rho +\sin \Theta \cos \delta \sin \rho \\ \sin \delta \sin \rho \end{array}\right).$$

Note that $\widehat{n}$ and $\widehat{\ell }$ are unit vectors.

## Supplementary information

Supplementary Information.
