# Supplementary material for: No Significant Effect of Coulomb Stress on the Gutenberg-Richter Law after the Landers Earthquake
Source: Sci Rep. 2020 Feb 19;10:2901. doi: 10.1038/s41598-020-59416-2 (PMC7031507; doi:10.1038/s41598-020-59416-2)
Supplement: Supplementary file 1 — Supplementary Information. [file 41598_2020_59416_MOESM1_ESM.pdf]

Supplementary Material -No Significant Effect of Coulomb Stress on the Gutenberg-Richter Law after the Landers Earthquake

Víctor Navas-Portella[1,2,3], Abigail Jiménez[4], and Álvaro Corral[1,2,5,6]

[1]Centre de Recerca Matemàtica, Edifici C, Campus Bellaterra, E-08193 Barcelona, Spain
[2]Barcelona Graduate School of Mathematics, Edifici C, Campus Bellaterra, E-08193 Barcelona, Spain
[3]Facultat de Matemàtiques i Informàtica, Universitat de Barcelona, Barcelona, Spain
[4]Departamento de Computación e Inteligencia Artificial, Universidad de Granada, Campus Ceuta, Cortadura del Valle s.n., E-51001 Ceuta, Spain
[5]Departament de Matemàtiques, Universitat Autònoma de Barcelona, E-08193 Barcelona, Spain
[6]Complexity Science Hub Vienna, Josefstädter Straβe 39, 1080 Vienna, Austria
[*]vnavas@crm.cat

ABSTRACT

We provide complementary figures and tables to the main text.

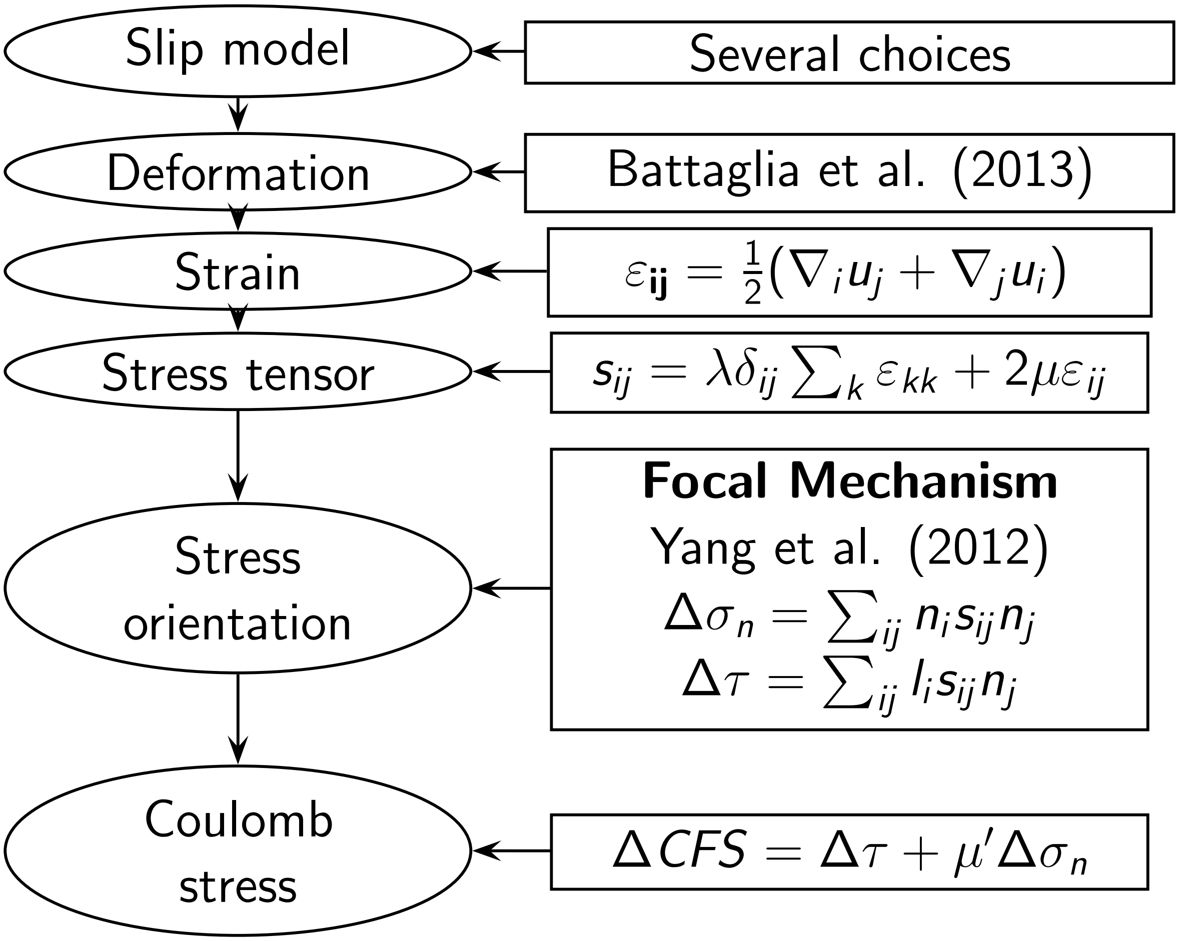

Figure 1. Flowchart summarizing the procedure to obtain the Coulomb stress on each aftershock fault plane from the slip model and the focal-mechanism catalog.

	N_{tot}	N	$b-$**value**	σ	p_{fit}
Overall	6027	560	$b_{all} = 0.909$	0.038	0.243 ± 0.004
$\mu' = 0.1$, $\Delta CFS > 0$	5083	488	$b_> = 0.925$	0.042	0.451 ± 0.005
$\Delta CFS < 0$	944	72	$b_< = 0.818$	0.096	0.162 ± 0.004
$\mu' = 0.2$, $\Delta CFS > 0$	5157	497	$b_> = 0.929$	0.042	0.297 ± 0.006
$\Delta CFS < 0$	870	63	$b_< = 0.783$	0.099	0.712 ± 0.006
$\mu' = 0.4$, $\Delta CFS > 0$	5213	509	$b_> = 0.927$	0.041	0.313 ± 0.005
$\Delta CFS < 0$	814	51	$b_< = 0.766$	0.107	0.861 ± 0.003
$\mu' = 0.6$, $\Delta CFS > 0$	5204	512	$b_> = 0.934$	0.041	0.315 ± 0.005
$\Delta CFS < 0$	823	48	$b_< = 0.710$	0.102	0.757 ± 0.004
$\mu' = 0.8$, $\Delta CFS > 0$	5138	509	$b_> = 0.920$	0.041	0.315 ± 0.005
$\Delta CFS < 0$	889	51	$b_< = 0.815$	0.114	0.632 ± 0.005

Table 1. Results of fitting the Gutenberg-Richter law to the Landers aftershocks, separating positive and negative Coulomb-stress increases as arising from the wald slip model, for different values of the effective friction coefficient μ' and $m_{min} = 3$. The overall case (with ΔCFS taking any sign) is also included and labelled as "all". Aftershocks correspond to the first 100 days after the Landers mainshock. Distance of aftershocks to the Landers rupture is restricted to be between 10 and 150 km. The $p-$value of the goodness-of-fit test is computed with 10^4 simulations and is denoted by p_{fit}. Its uncertainty corresponds to one standard deviation. We conclude that the value of μ' has little influence on $b_>$ and $b_<$.

Slip Model		N_{tot}	N	$b-$**value**	σ	p_{fit}
**wald**	$\Delta CFS > 0$	5474	525	$b_> = 0.936$	0.041	0.330 ± 0.005
	$\Delta CFS < 0$	553	35	$b_< = 0.637$	0.108	0.909 ± 0.003
	All	6027	560	$b_{all} = 0.909$	0.038	0.243 ± 0.004
**hernandez**	$\Delta CFS > 0$	5210	482	$b_> = 0.938$	0.043	0.439 ± 0.005
	$\Delta CFS < 0$	582	45	$b_< = 0.754$	0.112	0.255 ± 0.004
	All	5792	527	$b_{all} = 0.919$	0.040	0.231 ± 0.004
**bbcal**	$\Delta CFS > 0$	4059	337	$b_> = 0.993$	0.054	0.190 ± 0.004
	$\Delta CFS < 0$	773	54	$b_< = 0.854$	0.116	0.357 ± 0.005
	All	4832	391	$b_{all} = 0.971$	0.049	0.053 ± 0.002
**surfrup**	$\Delta CFS > 0$	5750	565	$b_> = 0.902$	0.038	0.245 ± 0.004
	$\Delta CFS < 0$	558	51	$b_< = 0.772$	0.108	0.864 ± 0.003
	All	6308	616	$b_{all} = 0.890$	0.036	0.239 ± 0.004

Table 2. Results of fitting the Gutenberg-Richter law to the Landers aftershocks, separating positive and negative Coulomb-stress increases computed in the nodal plain where it is maximum, for different Slip models, $\mu' = 0.4$ and $m_{min} = 3$. Aftershocks correspond to the first 100 days after the Landers mainshock. Distance of aftershocks to the Landers rupture is restricted to be between 10 and 150 km. The $p-$value of the goodness-of-fit test is computed with 10^4 simulations and is denoted by p_{fit}. Its uncertainty corresponds to one standard deviation. In no case the Gutenberg-Ricther law can be rejected.

Slip Model	z	p_{norm}	p_{perm}	ΔAIC
**wald**	2.600	0.009	0.006 ± 0.001	-3.460
**hernandez**	1.534	0.125	0.116 ± 0.003	-0.094
**bbcal**	1.081	0.280	0.312 ± 0.005	0.909
**surfrup**	1.136	0.256	0.256 ± 0.004	0.814

Table 3. Results of the statistical tests comparing b-values and magnitude distributions for positive and negative Coulomb-stress changes computed in the nodal plane where it is maximum, using different slip models and $\mu' = 0.4$. Values of $\Delta AIC = AIC_2 - AIC_1$ are also included. Same data as previous table. Columns 2 to 4: testing the null hypothesis that there is no difference between the $b-$values (i.e., $b_> = b_<$). In the first test, both asymptotic normality of the z statistic and a permutation test are used for the calculation of the $p-$value (labeled as p_{norm} and p_{perm}, respectively). In the latter case the number of permutations is 10^4, and the uncertainty of p_{perm} corresponds to one standard deviation.

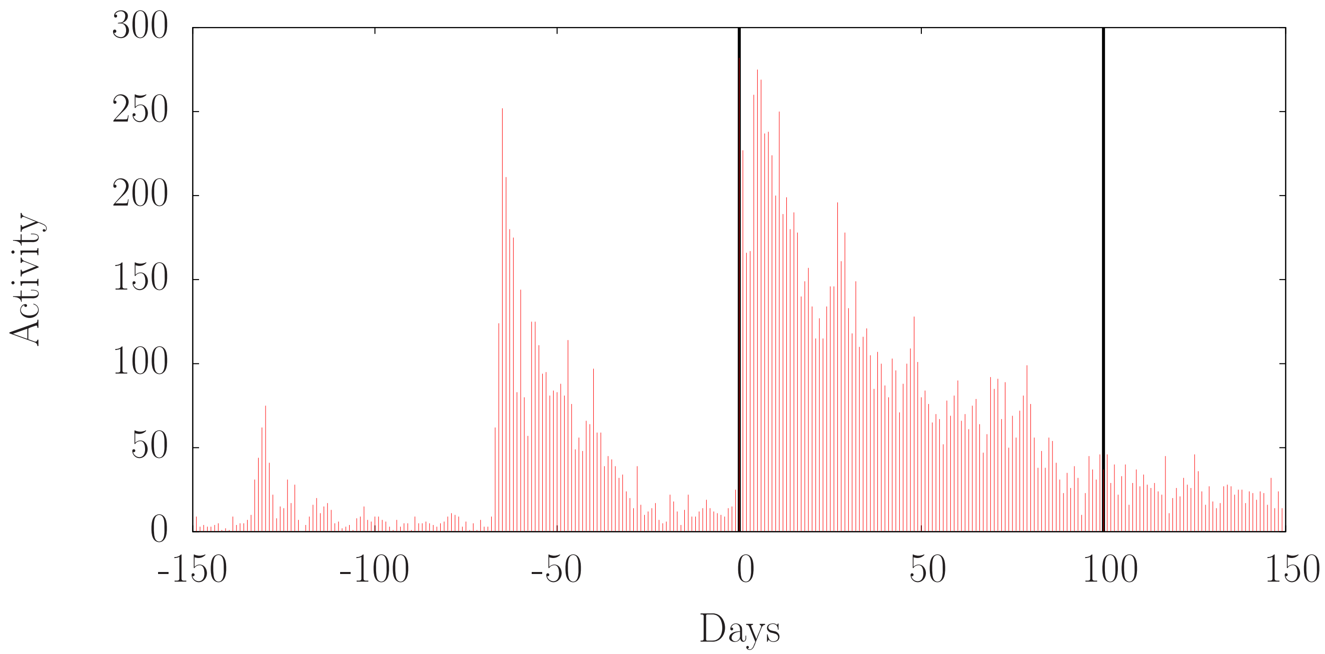

Figure 2. Number of earthquakes per day of any magnitude before and after the Landers mainshock in the YHS catalog in the area selected for our study. Black lines show the temporal window chosen in this work.

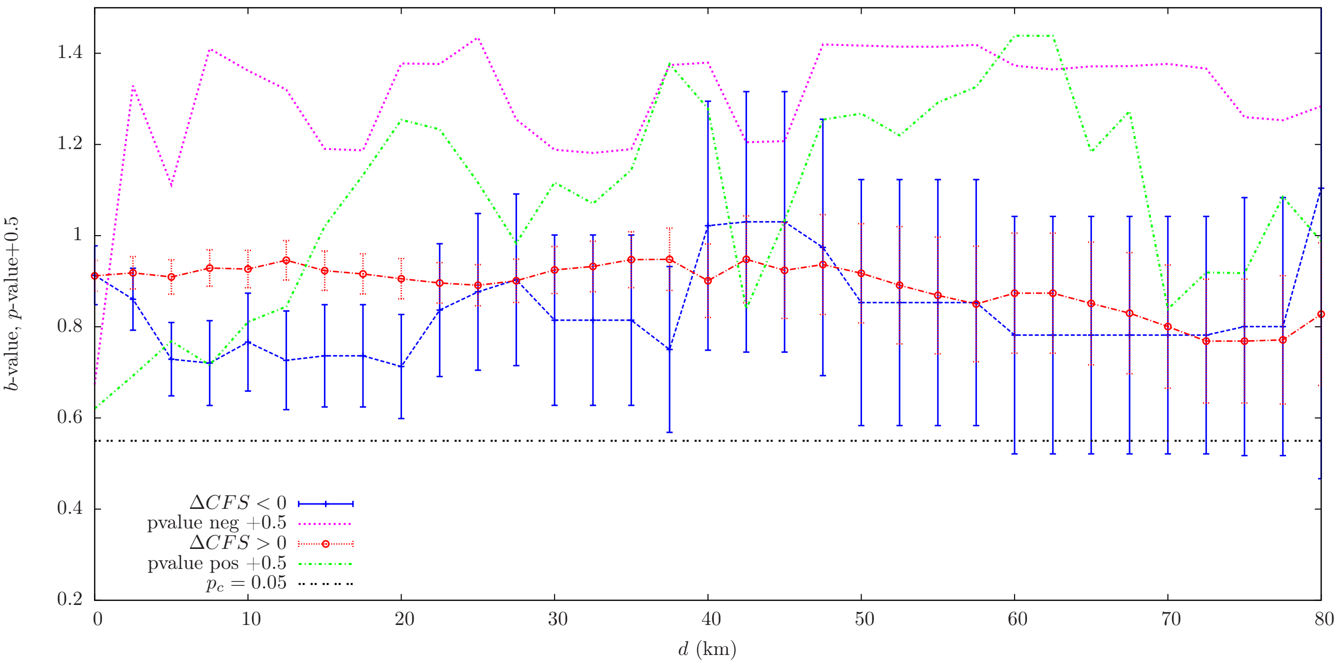

Figure 3. Dependence of the exponents $b_>$ and $b_<$ (blue and red respectively) on the distance of the events to the fault d for the wald slip model, with $m_{min} = 3$ and $\mu' = 0.4$. Green and purple dashed lines correspond to the p-values of the goodness-of-fit test shifted 0.5 for convenience. Horizontal black dashed line corresponds to the threshold p-value $p_c = 0.05$ shifted 0.5.

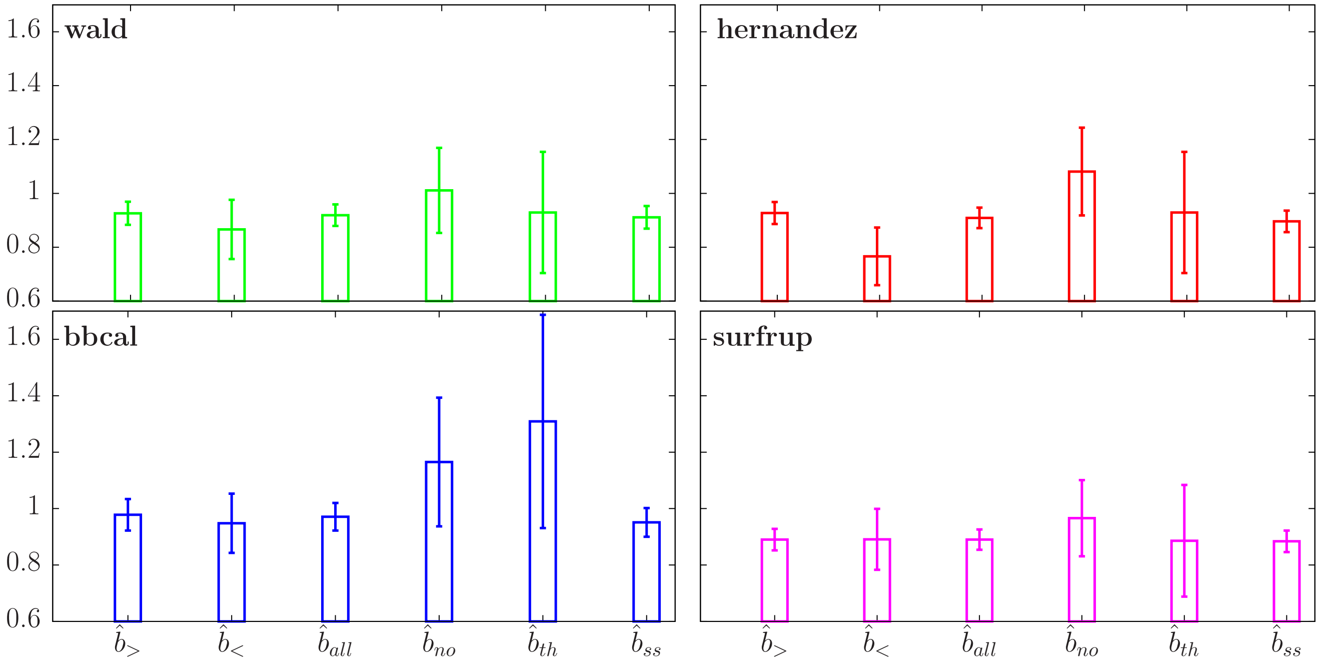

Figure 4. Visual representation of the results in Tables 1 and 3 in the main article. b-values of the Gutenberg-Richter law for Landers aftershocks, separating positive $\hat{b}_{>}$ and negative $\hat{b}_{<}$ Coulomb-stress increases as well as for the overall case $\hat{b}_{all}$ (with ΔCFS taking any sign) are shown for different slip models, $\mu' = 0.4$ and $m_{min} = 3$. Aftershocks correspond to the first 100 days after the Landers mainshock. Distance of aftershocks to the Landers rupture is restricted to be between 10 and 150 km for each slip model. b-values for aftershocks according to their focal mechanisms are also shown: normal $\hat{b}_{no}$, thrust $\hat{b}_{th}$ and strike-slip $\hat{b}_{ss}$.

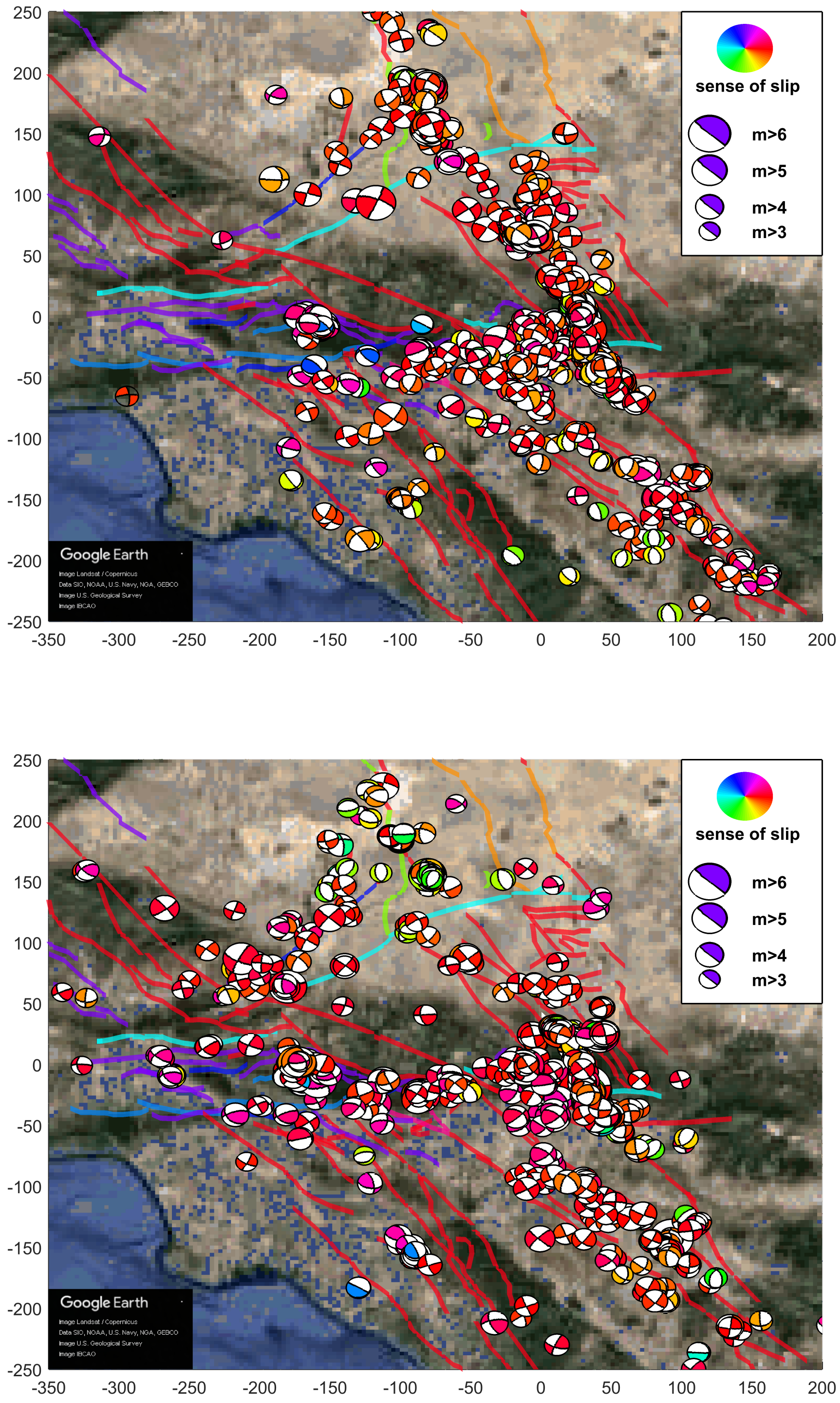

Figure 5. Focal mechanism representation for strike-slip Landers aftershocks, separated in terms of ΔCFS, as calculated from the hernandez slip model (time window of 100 days after the mainshock). Top: $\Delta CFS > 0$. Bottom: $\Delta CFS < 0$. Color scale represents the sense of slip and fault traces are also shown using the same color code. An area of 550×500 km is shown; aftershocks are restricted to $m \geq 3$ (for clarity sake). Both axes display distances with respect an arbitrary origin, in km.

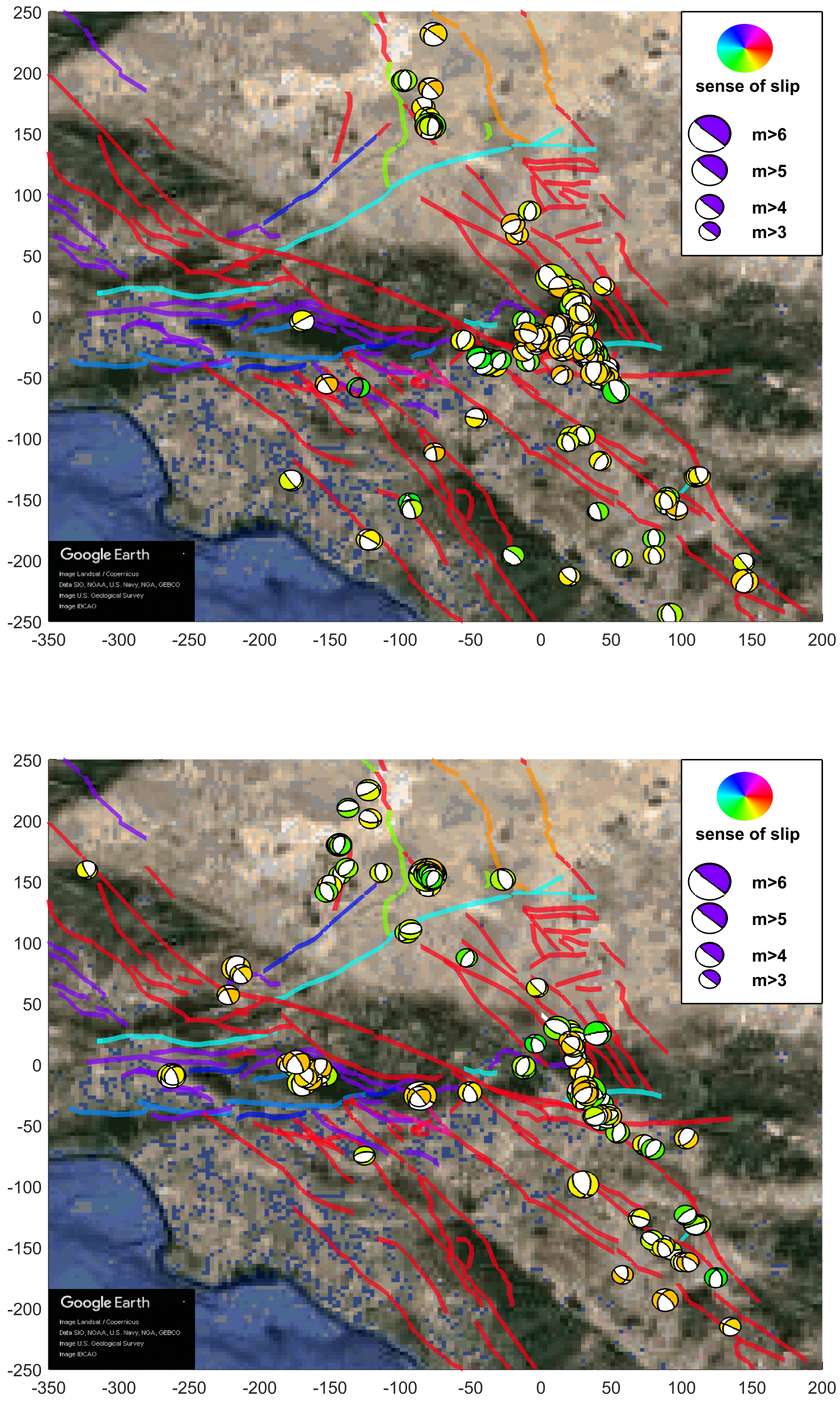

Figure 6. Focal mechanism representation for normal Landers aftershocks, separated in terms of ΔCFS, as calculated from the hernandez slip model (time window of 100 days after the mainshock). Top: $\Delta CFS > 0$. Bottom: $\Delta CFS < 0$. Color scale represents the sense of slip and fault traces are also shown using the same color code. An area of 550×500 km is shown; aftershocks are restricted to $m \geq 3$ (for clarity sake). Both axes display distances with respect an arbitrary origin, in km.

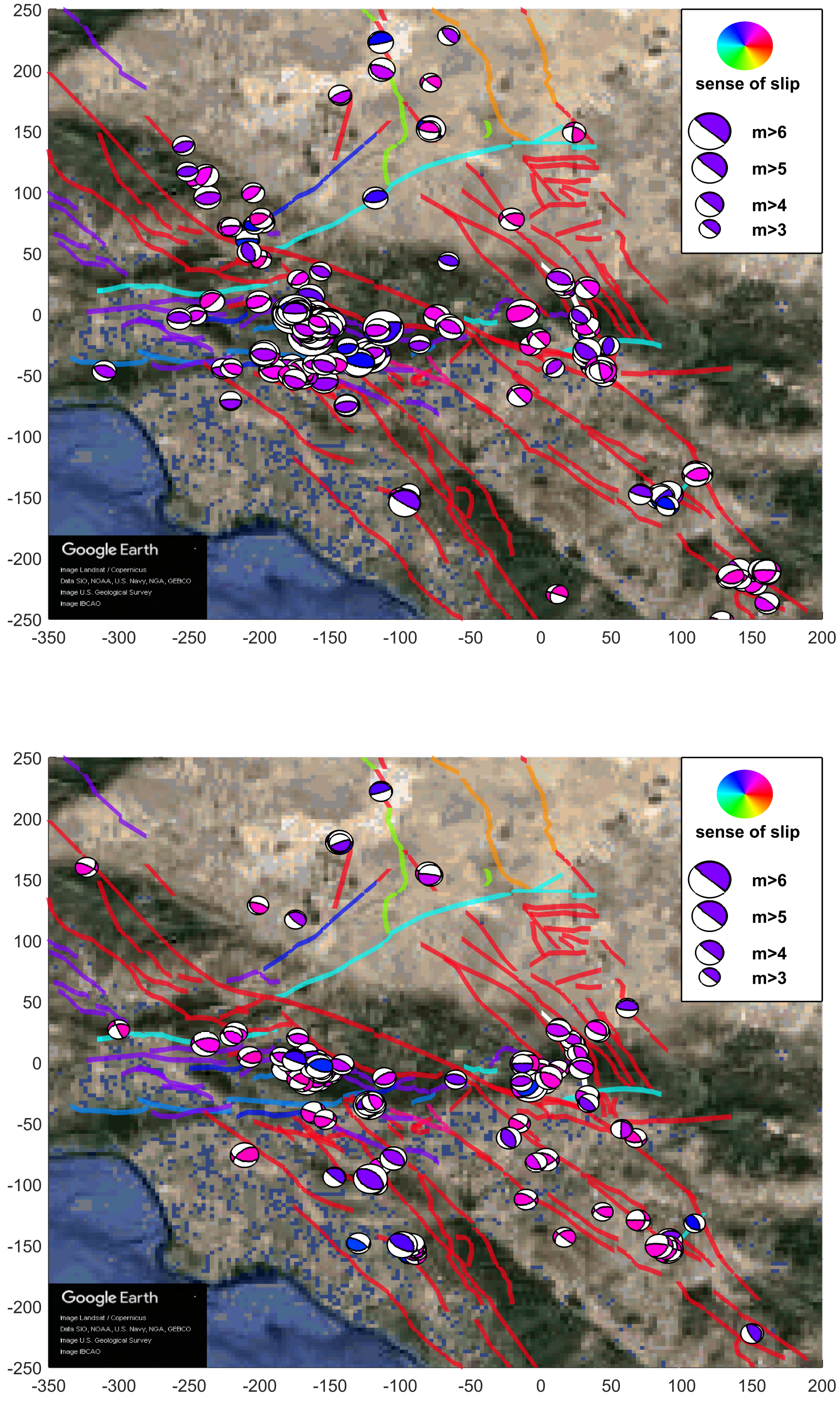

Figure 7. Focal mechanism representation for thrust Landers aftershocks, separated in terms of ΔCFS, as calculated from the hernandez slip model (time window of 100 days after the mainshock). Top: $\Delta CFS > 0$. Bottom: $\Delta CFS < 0$. Color scale represents the sense of slip and fault traces are also shown using the same color code. An area of 550×500 km is shown; aftershocks are restricted to $m \geq 3$ (for clarity sake). Both axes display distances with respect an arbitrary origin, in km.
